# Scoring clinical signs can help diagnose canine visceral leishmaniasis in a highly endemic area in Brazil

**DOI:** 10.1590/0074-02760160305

**Published:** 2017-01

**Authors:** Kleverton Ribeiro da Silva, Vitor Rosa Ramos de Mendonça, Kellen Matuzzy Silva, Leopoldo Fabrício Marçal do Nascimento, Antonio Ferreira Mendes-Sousa, Flaviane Alves de Pinho, Manoel Barral-Netto, Aldina Maria Prado Barral, Maria do Socorro Pires e Cruz

**Affiliations:** 1 Fundação Oswaldo Cruz-Fiocruz, Centro de Pesquisas Gonçalo Moniz, Salvador, BA, Brasil; 2 Universidade Federal do Piauí, Departamento de Morfofisiologia Veterinária, Teresina, PI, Brasil; 3 Universidade Federal do Piauí, Picos, PI, Brasil; 4 Universidade Federal da Bahia, Faculdade de Medicina, Salvador, BA, Brasil; 5 Instituto Nacional de Ciência e Tecnologia, Instituto de Investigação em Imunologia, São Paulo, SP, Brasil

**Keywords:** canine visceral leishmaniasis, clinical score, diagnosis, clinical signs

## Abstract

Canine visceral leishmaniasis (CVL) diagnosis is still a challenge in endemic areas with limited diagnostic resources. This study proposes a score with the potential to distinguish positive CVL cases from negative ones. We studied 265 dogs that tested positive for CVL on ELISA and parasitological tests. A score ranging between 0 and 19 was recorded on the basis of clinical signs. Dogs with CVL had an overall higher positivity of the majority of clinical signs than did dogs without CVL or with ehrlichiosis. Clinical signs such as enlarged lymph nodes (83.93%), muzzle/ear lesions (55.36%), nutritional status (51.79%), bristle condition (57.14%), pale mucosal colour (48.21%), onychogryphosis (58.93%), skin lesion (39.28%), bleeding (12.50%), muzzle depigmentation (41.07%), alopecia (39.29%), blepharitis (21.43%), and keratoconjunctivitis (42.86%) were more frequent in dogs with CVL than in dogs with ehrlichiosis or without CVL. Moreover, the clinical score increased according to the positivity of all diagnostic tests (ELISA, p < 0.001; parasite culture, p = 0.0021; and smear, p = 0.0003). Onychogryphosis (long nails) [odds ratio (OR): 3.529; 95% confidence interval (CI): 1.832-6.796; p < 0.001], muzzle depigmentation (OR: 4.651; 95% CI: 2.218-9.750; p < 0.001), and keratoconjunctivitis (OR: 5.400; 95% CI: 2.549-11.441; p < 0.001) were highly associated with CVL. Interestingly, a score cut-off value ≥ 6 had an area under the curve of 0.717 (p < 0.0001), sensitivity of 60.71%, and specificity of 73.64% for CVL diagnosis. The clinical sign-based score for CVL diagnosis suggested herein can help veterinarians reliably identify dogs with CVL in endemic areas with limited diagnostic resources.

American visceral leishmaniasis is a chronic parasitic zoonosis widespread in Latin America, with 90% of the cases occurring in Brazil, where it affects more than 3,300 individuals per year (Alvar et al. 2012). It is caused by a protozoan parasite *Leishmania infantum* (syn *L. chagasi*) transmitted by the bite of infected female sand flies of the genus *Lutzomyia*. This disease mainly affects malnourished children under 10 years of age, and it is commonly fatal if not treated early (Alvar et al. 2012). In endemic urban areas, domestic dogs are important hosts of the parasite, acting as an easy source of *Leishmania* infection for sand flies because of intense cutaneous parasitism (Dantas-Torres 2007). In addition, canine visceral leishmaniasis (CVL) is highly associated with cases of human disease (de Araújo et al. 2013).

Infected dogs can present different clinical features ranging from apparently healthy (asymptomatic dogs) to several characteristic signs (symptomatic dogs) such as lymphadenopathy, onychogryphosis, cutaneous lesions, alopecia, apathy, vomiting, fever, diarrhoea, polyuria, polydipsia, splenomegaly, and pale mucous membranes (Mancianti et al. 1988). Many studies have shown that the clinical signs of infected dogs are related to haematological and biochemical alterations as well as to antibody titres, parasite load, and infectivity to the sand fly vector (da Costa-Val et al. 2007, Manna et al. 2009, de Freitas et al. 2012, Manzillo et al. 2013, Ribeiro et al. 2013).

Diagnosis of CVL is a difficult task for veterinarians because of the following factors. First, the clinical signs are highly variable and are similar to those of other pathologies, such as ehrlichiosis (Tafuri et al. 2001, de Castro et al. 2004). Second, the histopathologic analysis is invasive, time-consuming, and expensive. Third, developing a 100% specific and sensitive diagnostic test is not possible (Ferrer 1999). In endemic regions with limited resources, the diagnostic procedure is even worse because serological or molecular methods are difficult to perform. In this context, a reliable clinical score based on clinical signs is needed to help CVL diagnosis and management in endemic regions. In Italy, a severity score based on CVL signs was proposed; however, it was not compared with the scores of CVL-negative dogs to predict its diagnostic capacity (Manna et al. 2009).

In this study, we propose a new scoring system based on frequently observed clinical signs in order to help diagnose CVL in regions with limited resources. In order to verify the efficacy of this clinical score, we applied it in a large sample of sick dogs (including CVL-positive and CVL-negative animals) that were brought to a reference veterinary hospital in a highly endemic, low-resource area in Brazil.

## MATERIALS AND METHODS

*Study design and dogs* - All dogs that were brought to the reference veterinary hospital of the Federal University of Piauí (UFPI) in Teresina, a city in the northeast of Brazil, between 2011 and 2012 underwent a careful clinical examination by trained veterinarians. The dogs were usually brought in when they showed clinical signs, and when the veterinarians from communities and small villages suspected them of having CVL. Moreover, sick, stray dogs collected by the Zoonosis Control Center following the Brazilian Zoonosis Control Program were also included. These animals were recruited because they were homeless dogs, and many of them were sick. The veterinary hospital at the UFPI is a regional reference centre for CVL, and all dogs showing clinical signs similar to leishmaniasis underwent routine clinical and laboratory tests for this pathology.

Fourteen different signs were evaluated in the dogs by observing the presence of signs attributable to *Leishmania* infection by using the following criteria:

*Systemic signs* - Attitude: active (0), apathetic (1); ectoparasites: absence (0), fleas (1), fleas and ticks (2); nutritional status: normal (0), thin (1), cachectic (2); lymph nodes: normal (0), enlarged (1); mucosal colour: normal (0), pale (1); bleeding: absence (0), presence (1).

*Cutaneous signs* - Bristles: good (0), regular (1), bad/opaque (2); muzzle/ear lesion: absence (0), presence (1); nails: normal (0), long/onychogryphosis (1); skin lesion: absence (0), presence (1), ulcer (2); muzzle depigmentation: absence (0), presence (1); alopecia: absence (0), presence (1).

*Ocular signs* - Blepharitis: absence (0), presence (1); keratoconjunctivitis: absence (0), serous (1), mucopurulent (2).

The clinical assessment was performed by three veterinarians who scored the signs according to the above criteria. In addition, all the individual scores were added to produce a total sign-based score ranging between 0 and 19, adapted from Manna et al. (2009).

In total, 443 mixed-breed adult male and female dogs of different ages (range, six months to 13 years) and breeds naturally infected with CVL were studied. The remaining dogs (sick dogs) were considered positive for CVL following a positive serological test and at least one positive parasitological test (CVL positive). Dogs with negative results on all three diagnostic tests were considered negative for CVL (CVL negative). CVL-positive dogs co-infected with *Ehrlichia canis* and/or *Babesia canis* were excluded from this study. In addition, a group of dogs infected by *E. canis* (and negative for CVL, n = 22) underwent clinical examination (based on the proposed score) and was included in this study.

*Diagnostic tests* - Blood was collected using jugular venipuncture, and samples of bone marrow were obtained using sternal puncture and lymph node aspiration (both tests for all dogs). Scrapings of skin lesions suggestive of CVL were also collected. The smears from these tissues were prepared and stained with Giemsa (Sigma Aldrich, St. Louis, MO, USA) stain and examined microscopically under a 100× objective for the presence of amastigotes. Bone marrow and lymph node aspirate was transferred to NNN-Schneider’s culture medium (Sigma-Aldrich), which was maintained at 25ºC and examined weekly for 30 days. Soluble *Leishmania* antigen was obtained from *Leishmania* spp. cultivation using 1 × 10^10^ promastigotes (Souza et al. 2013) to detect IgG anti-*Leishmania* antibodies by using an ELISA (Badaró et al. 1986). The serum titration used was 1:400, and the cut-off was calculated for each plate as the mean of three negative sera ± 2 standard deviations. Diagnostic CVL tests, including ELISA, parasite culture, or smear, were performed for all dogs. Dogs were considered positive for CVL following a positive ELISA (highly sensitive method) and positive parasite culture or smear (highly specific methods). Dogs considered negative for CVL were those with negative results for all three diagnostic tests.

*E. canis* and *B. canis* infections were diagnosed through nested polymerase chain reaction (PCR) and PCR, respectively, via protocols routinely used in the Animal Pathology Laboratory of the UFPI by using DNA from peripheral blood (Wen et al. 1997, Jefferies et al. 2007). Detection of *E. canis* was based on the 16S rRNA gene and *B. canis* was based on the 18S rRNA gene.

*Ethics statement* - This study was approved by the Institutional Review Board of the UFPI, under protocol number 021/12. In addition, the animal care protocols used in this study adhered to the guidelines regulated by the Brazilian College of Animal Experimentation.

*Data analysis* - In the exploratory analysis of the data, frequency tables were constructed and the Chi-square or Fisher’s exact tests were applied to evaluate the association between the qualitative variables (sex and breed). The severity of clinical signs was treated as an ordinal variable. The quantitative variables (total score and score based on statistically different signs) were tested for Gaussian distribution within the total sample by using the D’Agostino-Pearson omnibus normality test. None of the variables showed a normal distribution (p < 0.0001 for all; thus, they did not pass the normality test), and hence, non-parametric tests were used. In this context, the Mann-Whitney test was used to assess the differences between two groups. Heat maps and hierarchical cluster analyses using Ward’s method were performed to better understand the association of signs in all sick dogs and according to CVL status. Univariate logistic regression analysis was performed to test the associations between the presence or absence of each clinical sign and clinical score cut-off values with CVL positivity. Receiver operator characteristic (ROC) curves with C-statistics were used to establish the threshold value of clinical scores that could discriminate between positive and negative CVL.

In order to understand how the signs associate with each other, network analyses based on clinical signs and represented by statistically significant correlations were performed for all sick and CVL-positive dogs. Correlations between all quantitative variables were analysed with the Spearman test by using the JMP 11.0 software (SAS, Cary, NC, USA), and these correlations were used to build the networks represented by the association between signs. The links between the signs represent only the statistically significant correlations (p < 0.05). The complexity of the networks was estimated from the density of interactions of each network, which could be measured using the following equation: density (D) = L/(N (N-1)/2), where L is the number of observed edges (*i.e.*, Spearman correlations with p < 0.05) and N is the total number of nodes in the network; the density could range between 0 (no edges in the network) and 1 (all possible edges present). Network figures were customised using the Pathways Analysis software (Ingenuity Systems, Redwood City, CA, USA) and Adobe Illustrator (Adobe Systems Inc., Seattle, WA, USA) The statistical analyses were performed using the programs GraphPad Prism 6.0 (GraphPad Software Inc., USA), IBM SPSS Statistics for Windows/Macintosh, Version 22.0 (IBM Corp., Armonk, NY, USA), and JMP 11.0 (SAS). A p value lower than 0.05 was considered statistically significant.

## RESULTS

*Baseline characteristics* - Of the 443 dogs screened, 178 co-infected with *L. infantum* and *E. canis* or *B. canis* were excluded from this study (n = 120 and 58, respectively). Finally, 265 sick dogs irrespective of sex and breed were recruited; 89 (33.58%) were originally from the veterinary hospital and 176 (66.42%) from the Brazilian Zoonosis Control Program. The percentage of male and female animals was very similar (47.55% males; Table I). No differences were seen in the sex or breed of dogs relative to the presence or absence of CVL (p = 1.000 and p = 0.539, respectively). Reproductive status was not recorded in this study. The distribution of breeds among all the sick dogs and according to CVL status is described in detail in Table I.

**TABLE I:** Baseline characteristics of sick dogs recruited in this study

	CVL positive	CVL negative	All dogs	p value*
	n = 56	n = 129	n = 265	
Male n(%)	26 (46.43)	60 (46.51)	126 (47.55)	1.000**
Breed n(%)				
Dalmatian	0 (0.00)	1 (0.77)	2 (0.75)	0.539***
Brazilian mastiff	0 (0.00)	1 (0.77)	3 (1.13)	
German Shepherd	1 (1.79)	2 (1.55)	5 (1.89)	
Pinscher	2 (3.57)	3 (2.33)	7 (2.64)	
Pit bull	2 (3.57)	4 (3.10)	9 (3.40)	
Poodle	2 (3.57)	9 (6.98)	14 (5.28)	
Rottweiler	1 (1.79)	3 (2.33)	5 (1.89)	
Yorkshire	0 (0.00)	0 (0.00)	2 (0.75)	
Mongrel	46 (82.14)	94 (72.87)	201 (75.85)	
Others	2 (3.57)	12 (9.30)	17 (6.42)	

*: p value was calculated by comparing the canine visceral leishmaniasis (CVL)-positive and CVL-negative subjects; **: Fisher’s exact test was used; ***: Chi-square test was used, and breeds with less than two dogs in each group were excluded.

The frequency of all the clinical signs recorded in the score in the different study groups (CVL-positive, CVL-negative, and *E. canis*-infected dogs) are described in Table II. As expected, the dogs with CVL had an overall higher positivity for the majority of clinical signs than did dogs without CVL or with ehrlichiosis (Table II). Clinical signs such as enlarged lymph nodes (p < 0.0001), muzzle/ear lesions (p < 0.0001), nutritional status (p < 0.0001), bristle condition (p = 0.0001), pale mucosal colour (p = 0.0202), onychogryphosis (p < 0.0001), muzzle depigmentation (p = 0.0002), alopecia (p = 0.0129), blepharitis (p = 0.0159), and keratoconjunctivitis (p = 0.0009) were more frequent in dogs with CVL than in those with ehrlichiosis (Table II). To confirm whether these clinical signs were more important in CVL cases, the signs were compared with those of sick dogs without CVL and with ehrlichiosis. Thus, it was seen that dogs with ehrlichiosis had less enlarged lymph nodes (p < 0.0001), less muzzle/ear lesions (p = 0.0022), less onychogryphosis (p = 0.0274), and better nutritional status (p = 0.0274) and bristle condition (p = 0.0035) (Table II).

**TABLE II:** Frequency of clinical signs in dogs with canine visceral leishmaniasis (CVL), ehrlichiosis, and without CVL

Clinical signs n(%)	1	2	3	p value 1 vs. 2	p value 1 vs. 3	p value 2 vs. 3
	VL positive	VL negative	*E. canis*			
	n = 56	n = 129	n = 22			
Apathy*	3 (5.36)	4 (3.10)	1 (4.54)	0.4341	1.000	0.5502
Nutritional status**						
Thin	26 (46.43)	35 (27.13)	1 (4.54)	0.0023	< 0.0001	0.0274
Cachectic	3 (5.36)	0 (0.00)	0 (0.00)			
Enlarged lymph nodes*	47 (83.93)	88 (68.22)	5 (22.73)	0.0308	< 0.0001	< 0.0001
Pale*	27 (48.21)	46 (35.66)	4 (18.18)	0.1404	0.0202	0.1425
Bleeding*	7 (12.50)	4 (3.10)	0 (0.00)	0.0194	0.1815	1.000
Bristles**						
Regular	23 (41.07)	43 (33.33)	1 (4.54)	0.1100	0.0001	0.0035
Bad/opaque	9 (16.07)	11 (8.53)	0 (0.00)			
Muzzle/ear lesion*	31 (55.36)	36 (27.91)	0 (0.00)	0.0005	<0.0001	0.0022
Onychogryphosis*	33 (58.93)	37 (28.68)	2 (9.09)	0.0001	<0.0001	0.0650
Skin lesion***						
Presence	16 (28.57)	19 (14.73)	3 (13.64)	0.0162	0.0695	0.5727
Ulcer	6 (10.71)	6 (4.65)	0 (0.00)			
Muzzle depigmentation*	23 (41.07)	17 (13.18)	0 (0.00)	< 0.0001	0.0002	0.1352
Alopecia*	22 (39.29)	29 (22.48)	2 (9.09)	0.0308	0.0129	0.2511
Blepharitis*	12 (21.43)	8 (6.20)	0 (0.00)	0.0039	0.0159	0.6039
Keratoconjunctivitis**						
Serous	21 (37.50)	12 (9.30)	1 (4.54)	<0.0001	0.0009	0.4693
Mucopurulent	3 (5.36)	4 (3.10)	0 (0.00)			

*: Fisher’s exact test was used; **: positive signals were combined and analysed using Fisher’s exact test; ***: Chi-square test was used.

**Fig. 1:**
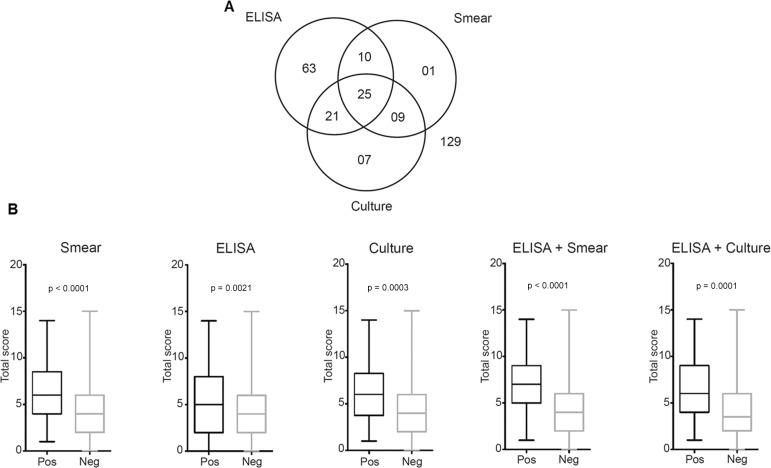
: canine visceral leishmaniasis (CVL) diagnostic tests and clinical signs. ELISA, smear, and culture tests for CVL were performed for all dogs brought to a reference veterinary hospital in the endemic area (A). Total clinical scores (range, 0-19) were higher in dogs with positive results on ELISA, culture, and smear tests and for the combinations of ELISA + culture or ELISA + smear tests (B). Data were compared using the Mann-Whitney test (B).

*Diagnostic tests and clinical signs* - ELISA, cultures, and smears were performed in all dogs. The positivity for each and the association of tests are described in Fig. 1A. Of the 265 dogs, 129 (48.68%) tested negative for all tests and were considered negative for CVL. Fifty-six dogs (21.13%) had positive ELISA and positive parasite culture results (n = 46) or/and positive parasite smear results (n = 35), and were considered positive for CVL (Fig. 1A). The next objective was to determine whether the total score value (range, 0-19), which combined all the signs, differed according to the results of the diagnostic tests (Fig. 1B). The sign-based score was higher for the positive results of all three individual diagnostic tests (ELISA, p < 0.001; parasite culture, p = 0.0021; and smear, p = 0.0003) and their combined results (ELISA and parasite culture, p < 0.0001; ELISA and parasite smear, p < 0.0001) than for the negative results of these tests (Fig. 1B).

**Fig. 2:**
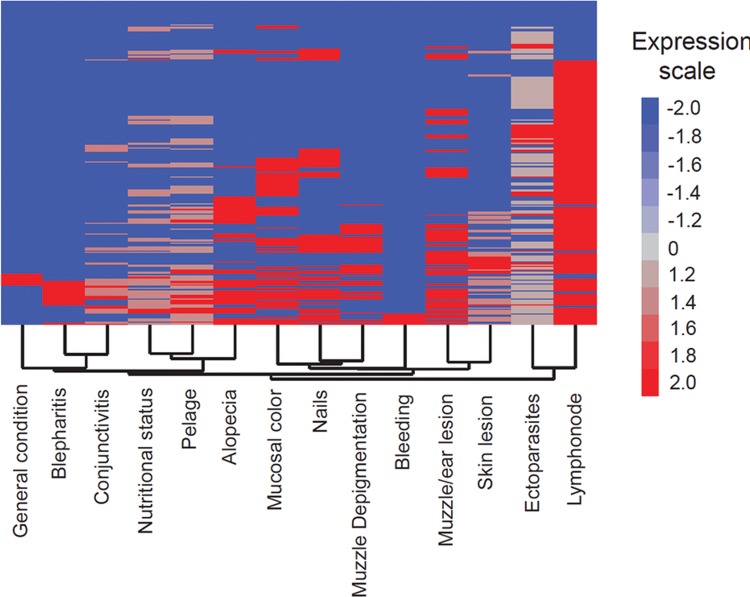
: heat map analysis of all sick dogs. Fourteen different signs were evaluated according to the proposed clinical score, and a two-way hierarchical cluster analysis (Ward’s method) was performed to identify patterns of associations between different signs among all the dogs. The colours show the fold variation from the median values (log transformed) calculated for each sign as represented by the expression scale.

A heat map analysis was performed for identifying clusters based on the signs of sick dogs (Fig. 2). Overall, the presence of some signs was more frequent in sick dogs with CVL, as illustrated by lymph node enlargement, presence of ectoparasites, alterations in the bristles, and muzzle/ear lesions (Fig. 2). In addition, organ-related signs from the proposed score (systemic, cutaneous, and ocular groups) had a general trend for clustering. Clusters were observed between the ocular signs (keratoconjunctivitis and blepharitis) and between the cutaneous signs (skin lesion and muzzle/ear lesion, for example), demonstrating that these signs were characteristic of sick dogs from this endemic area (Fig. 2).

**Fig. 3:**
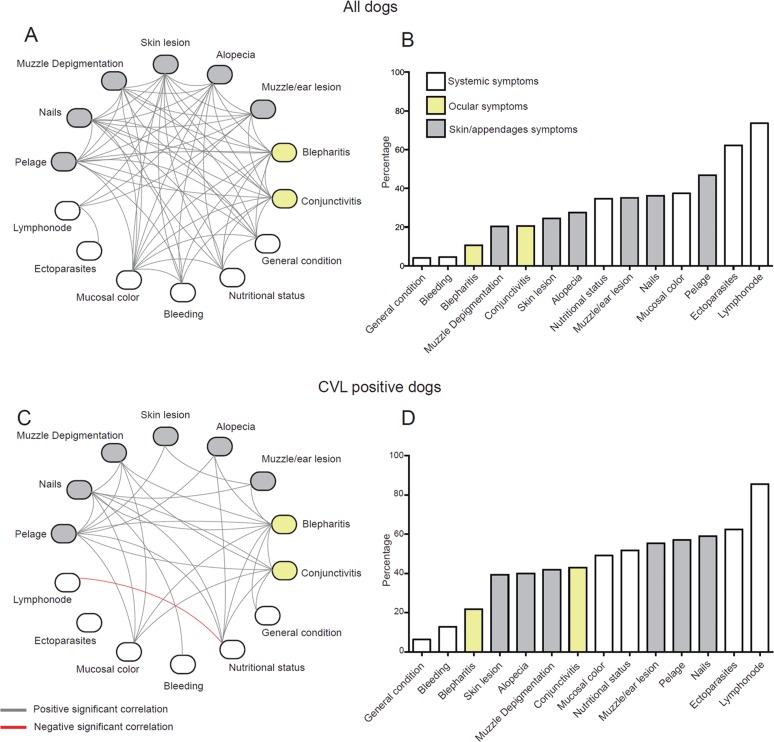
: networking the clinical signs of dogs with canine visceral leishmaniasis (CVL). The network analysis (interactome) shows statistically significant correlations (p < 0.05) between all the signs recorded for all sick dogs (A) and CVL-positive dogs (C). Data were analysed using Spearman rank tests. Yellow-coloured items are ocular signs, grey icons are skin/appendage signs, and white entries are systemic signs. Grey and red lines represent significant positive and negative correlations, respectively. The percentages of positivity for each sign are illustrated for all dogs (B) and CVL-positive dogs (D).

*Networking the clinical signs according to CVL status* - A higher complexity, measured by the network density (D = 0.346), was observed in all dogs when compared with the CVL-positive dogs (D = 0.165) (Fig. 3A, 3C, respectively); however, this could be attributed to the different sample sizes. The majority of signs (except lymph node size and ectoparasite presence) had a high level of association in all dogs brought into the reference veterinary hospital (Fig. 3A). The percentage of positivity for each sign for all dogs is illustrated in Fig. 3B. In the CVL-positive dogs, half of the signs (keratoconjunctivitis, mucosal colour, muzzle depigmentation, nutritional status, nails, bristles, and blepharitis) had the highest number of connections, suggesting their important role in the final clinical profile. The only significant negative correlation was between the lymph node size and nutritional status variables (Fig. 3C). The percentage of positivity for each sign in the CVL-positive dogs is demonstrated in Fig. 3D. The results showed that the overall positivity for the clinical signs was slightly more expressed in CVL-positive dogs than in all the sick dogs, and that the relative distributions of some signs differed between the two groups, as illustrated by serous or mucopurulent keratoconjunctivitis (42.86% and 20.65%, p = 0.001, Fig. 3D, B, respectively).

**Fig. 4:**
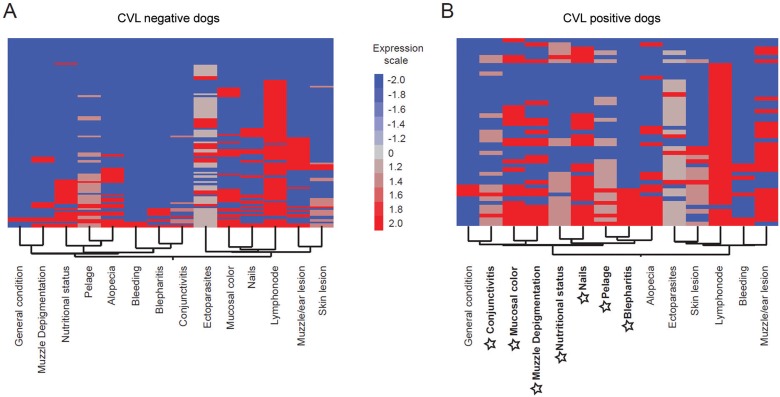
: heat map analysis of the clinical signs of dogs according to canine visceral leishmaniasis (CVL) status. Two-way hierarchical cluster analysis (Ward’s method) was performed to identify patterns of associations between different signs for CVL-negative dogs (A) and CVL-positive dogs (B). The colours show the fold variation from the median values (log transformed) calculated for each sign. Highlighted in bold and with star symbols are those signs with the highest association observed in CVL-positive dogs in Fig. 3C.

Heat map analyses according to the different signs were performed for CVL-negative and CVL-positive dogs (Fig. 4A-B, respectively). The results showed that the majority of signs were expressed more highly in the CVL-positive dogs than in the CVL-negative dogs. Moreover, the signs with the highest connectivity demonstrated in Fig. 3C also clustered in the CVL-positive heat map (highlighted in Fig. 4B) with smaller clusters between the following pairs of signs: mucosal colour and muzzle depigmentation; nutritional status and nails; and bristles and blepharitis (Fig. 4B).

**Fig. 5:**
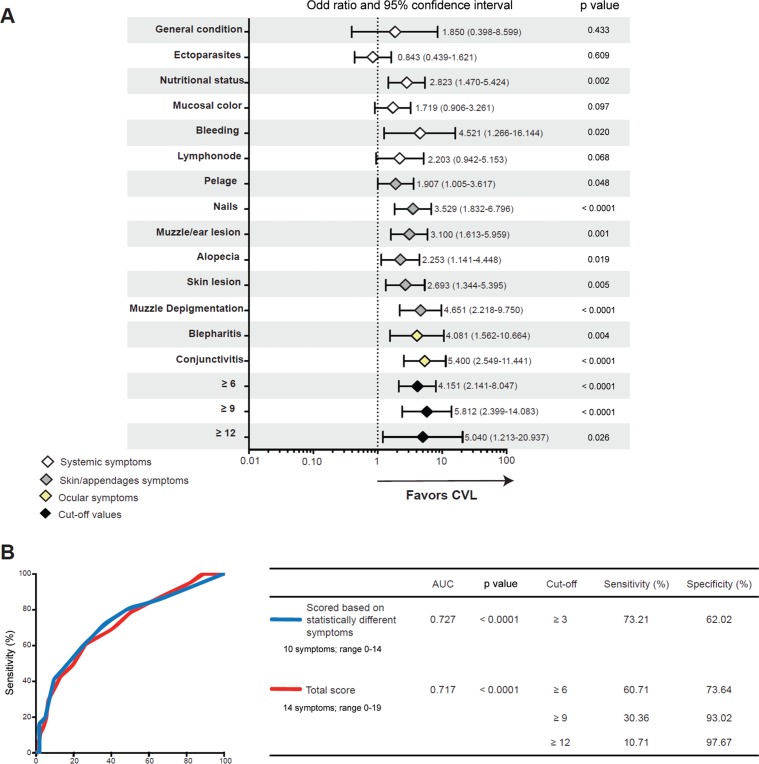
: association of clinical signs and clinical scores with canine visceral leishmaniasis (CVL). Univariate logistic regression analyses were conducted to estimate the association of each sign [based on median values or cut-off values (≥ 6, ≥ 9, and ≥ 12)] with CVL (A). Yellow-coloured items are ocular signs, grey icons are skin/appendage signs, white items are systemic signs, and black ones represent random cut-off values for the total clinical score (range, 0-19). Receiver operator characteristic (ROC) curves of the total score (red line) and the score based on statistically significant different signs (based on the analysis in A; blue line) for discriminating between dogs with and without CVL are shown in B. AUC, area under the curve. The cut-off values for the scores were established using C-statistics and are shown in the table (B). The odds ratios (OR), respective 95% confidence intervals (95% CIs), and p values are shown in each panel.

*Association between clinical profile and CVL status* - Ten of the 14 signs seemed to favour CVL, with special influences of onychogryphosis (long nails) (OR: 3.529; 95% CI: 1.832-6.796; p < 0.001), muzzle depigmentation (OR: 4.651; 95% CI: 2.218-9.750; p < 0.001), and keratoconjunctivitis (OR: 5.400; 95% CI: 2.549-11.441; p < 0.001) (Fig. 5A). Apathy (attitude), presence of ectoparasites, pale mucosal colour, and lymph node enlargement did not show any influence on CVL (p = 0.433, p = 0.609, p = 0.097, and p = 0.068, respectively; Fig. 5A). The cut-off values (≥ 6, ≥ 9, and ≥ 12) for the total clinical score (range, 0-19) established by using the ROC curve and C-statistics analyses (Fig. 5B) were also imputed in the regression model. The cut-off values ≥ 6 (OR: 4.151; 95% CI: 2.141-8.047; p < 0.001) and ≥ 9 (OR: 5.812; 95% CI: 2.399-14.083; p < 0.001) had almost similar performance in distinguishing between the CVL-positive and CVL-negative animals, and the cut-off value ≥ 12 had a lower precision (with a higher CI) than did the other described cut-off values (OR: 5.040; 95% CI: 1.213-20.20937; p = 0.026) (Fig. 5A).

The total clinical score (14 signs; range, 0-19) and a score based on the statistically different signs (10 signs; range, 0-14) shown in Fig. 5A had considerable power to distinguish between the CVL-positive and the CVL-negative dogs (Fig. 5B). The total score had an area under the curve (AUC) of 0.717 (p < 0.0001), and the cut-off value of ≥ 6 was the one with the best sensitivity for association (60.71%) and specificity (73.64%) to discriminate the CVL-positive and CVL-negative dogs (Fig. 5B). With the higher cut-off values (≥ 9 and ≥ 12), the specificity increased (93.02% and 97.67%, respectively) despite the decrease in sensitivity (30.36% and 10.71%, respectively). Furthermore, the score based on statistically different signs had an AUC of 0.727 (p < 0.0001), and the cut-off value ≥ 3 had a sensitivity and specificity of 73.21% and 62.02%, respectively, to discriminate animals according to CVL status (Fig. 5B).

The score proposed herein was applied in dogs with ehrlichiosis (but without CVL) in order to assess the ability of this score to test the specificity to CVL diagnosis. The clinical signs observed in the subjects with ehrlichiosis are described in Table II. With a cut-off value ≥ 3, the total score had an AUC of 0.9391 (p < 0.0001), and a sensitivity and specificity of 85.71% and 86.36%, respectively, to distinguish the CVL-positive dogs from those with ehrlichiosis.

## DISCUSSION

The serological and molecular diagnostic methods for CVL may be unavailable in endemic areas with limited resources, and in such areas, the diagnosis may be based on only clinical signs. In the present study, dogs that tested positive for CVL with different diagnostic methods (ELISA, parasite smear, or culture) had a higher clinical score than did CVL-negative sick dogs. The cut-off value > 6 obtained from the clinical score proposed herein performed well in distinguishing CVL-positive dogs from CVL-negative sick dogs with 60.71% and 73.64% sensitivity and specificity, respectively. Gouvêa et al. (2016), who conducted a study in the same area, found a cut-off value > 2 for a model based on the clinical score only (sensitivity = 75.3%; specificity = 65.9%) and a cut-off value > 9 for an association of IFAT + clinical score (sensitivity = 86.5%; specificity = 70%), thereby showing it was the best cut-off value for detecting the most infectious dogs and for disease control.

The clinical signs of CVL are important for diagnosis, and the clinical score based on sick dogs’ clinical signs proposed herein was higher in subjects who tested positive for CVL on different diagnostic tests, suggesting it can help in diagnosis depending on the cut-off value chosen. A group of clinical signs of *Leishmania* infection (keratoconjunctivitis, mucosal colour, muzzle depigmentation, nutritional status, nails, bristles, and blepharitis) was highly associated and clustered in the heat map results. Furthermore, the sole presence of onychogryphosis, muzzle depigmentation, or keratoconjunctivitis correlated highly with leishmaniasis. Manna et al. (2009) also created a severity clinical score that was associated with parasite load in CVL; however, this severity score was not compared with those of sick dogs without CVL to establish its diagnostic power. Other studies have demonstrated a model based on a scoring system combining the clinical signs and serological (IFAT) results as a tool to help in CVL control strategies (Proverbio et al. 2014, Gouvêa et al. 2016).

CVL diagnosis is still a challenge for veterinarians because of the lack of a perfect diagnostic test for this condition (Solano-Gallego et al. 2011). In the present study, CVL-positive dogs were diagnosed by both a positive serological test (ELISA) and a positive parasitological test (parasite culture or smear) to confirm CVL diagnosis by using high-sensitivity and high-specificity methods, respectively. CVL diagnoses based only on anti-*Leishmania* antibody detection are not satisfactory as they do not discriminate between the disease and an asymptomatic condition; moreover, the antibodies cross-react with other pathogens (Sarkari et al. 2005, Zanette et al. 2014). For example, the conventional serological methods using crude antigen to detect antibodies against *Leishmania* spp. show cross-reactivity with *Trypanosoma cruzi* because both parasites share similar antigenic epitopes owing to their phylogenetic similarity (Zanette et al. 2014). Moreover, dogs infected with *T. cruzi* have not yet been detected in Teresina, PI. These aspects may have contributed to the different sensitivity and specificity results obtained in the studies that used serological methods for detecting CVL (de Arruda et al. 2016).

In both sick and CVL-positive dogs, lymph node enlargement was the most common sign. In several case series, lymphadenomegaly was reported as the most frequent clinical sign in dogs with CVL (Ciaramella et al. 1997, Moreno & Alvar 2002, Amusategui et al. 2003, Manna et al. 2009). However, lymph node enlargement is a frequent response to infectious agents, and it is not specific to CVL (Mylonakis et al. 2011, Rodríguez-Morales et al. 2011, Salem & Farag 2014).

Keratoconjunctivitis and ocular signs in general are commonly reported in several case series with similar frequencies (Ferrer 1999, Amusategui et al. 2003, Manna et al. 2009, de Freitas et al. 2012, Manzillo et al. 2013). In the present report, 42.86% and 21.82% of dogs with CVL had keratoconjunctivitis and blepharitis, respectively. Intriguingly, the clinical spectrum of CVL may vary according to the phase of the disease, the dog’s immunity status, and previous specific therapies; thus, these features should be considered during CVL diagnosis (Ciaramella et al. 1997, Manzillo et al. 2013).

In the network analysis, we observed that some clinical signs (keratoconjunctivitis, mucosal colour, muzzle depigmentation, nutritional status, nails, bristles, and blepharitis) were more associated with CVL positivity than were other signs. Moreover, these signs clustered in the heat map analysis, indicating their important role in predicting CVL status. Ocular signs (*i.e.*, blepharitis and keratoconjunctivitis), cutaneous signs (*i.e.*, onychogryphosis and opaque bristles), and systemic signs (*i.e.*, pale mucous membrane and weight loss) have been extensively described in CVL clinical studies (Ciaramella et al. 1997, Amusategui et al. 2003, Manna et al. 2009, de Freitas et al. 2012, Manzillo et al. 2013). Cutaneous findings are very common in dogs with CVL, and the presence of skin lesions (including ulcers), onychogryphosis, alterations in the bristles, dermatitis, and alopecia should be carefully considered in sick dogs from endemic areas (Ciaramella et al. 1997, Ferrer 1999).

Among the 14 different clinical signs that comprise the clinical score proposed in this study, many could be used to differentiate CVL-positive dogs from other sick dogs, especially the presence of keratoconjunctivitis, onychogryphosis, and muzzle depigmentation in the CVL-positive dogs. Mancianti et al. (1988) proposed classifying dogs with CVL into asymptomatic, oligosymptomatic, and symptomatic cases, with the presence of onychogryphosis, keratoconjunctivitis, and cutaneous alterations being the clinical signs expected in symptomatic dogs. Interestingly, chronic cutaneous changes and ocular lesions were also signs that characterised dogs with severe CVL (Ciaramella et al. 1997). In addition, the cut-off values for the clinical score proposed herein performed well (AUCs higher than 70%) in distinguishing CVL-positive dogs from other sick dogs (with different diseases). Higher cut-off values had better specificity for CVL diagnosis because dogs with elevated clinical scores had more signs of this disease; although high cut-off values lacked sensitivity, they could be used with good reliability for confirming CVL in severely sick dogs in endemic areas.

In this study, 17 dogs had positive smear and/or culture test results and negative ELISA results for CVL (Fig. 1A). Notably, when the clinical scores of the CVL-positive dogs (based on this study criteria) were compared to those with only positive smear and/or culture test results, the latter were found to have a lower score (p = 0.0057). This could probably be because these dogs were in the initial stages of infection and had not yet had a clinical response to leishmaniasis.

In this study, we had excluded CVL-positive dogs co-infected with *E. canis* and *B. canis*, which can produce clinical findings similar to CVL (Ciaramella et al. 1997). Some studies have demonstrated that dogs with co-infection presented more severe clinico-pathological abnormalities and were frequently misdiagnosed in routine veterinary practices (Andrade et al. 2014). Thus, we have applied our clinical model only in dogs with CVL and ehrlichiosis. When the clinical score was applied in dogs infected with *E. canis*, we observed a great discriminatory power to distinguish dogs with CVL from those with ehrlichiosis, suggesting that the ability of this score to detect CVL when *E. canis* infection was not previously excluded. Dogs with other diseases requiring potential differential diagnosis for CVL were also included in the present study. Dogs with trypanosomiasis caused by *T. cruzi* have a general lymphadenopathy (Barr et al. 1991), which is a usual clinical sign found in all dogs in this study. Furthermore, severe seborrhoea, focal or diffuse alopecia, pustules, and blemishes are frequently observed in 12% to 23% of dogs with canine atopic dermatitis and could be found in the CVL-negative sick dogs (Hensel et al. 2015). Similarly, bacterial skin diseases, superficial and deep folliculitis, and keratinisation disorders may also be confused with clinical signs of CVL and should be considered in the CVL-negative dogs (Cardoso et al. 2011) .

This study proposes a clinical sign-based score for CVL diagnosis that can help veterinarians reliably identify dogs with CVL. Although there is no evidence supporting dog culling for CVL control, culling is still practiced in many endemic areas; this makes determining a correct diagnosis of CVL even more important (Costa 2011). The clinical score proposed herein is a guide for veterinarians mainly from highly endemic areas with limited diagnostic resources. Moreover, this clinical score can help confirm the accuracy of some diagnostic test results (*i.e.*, serological methods). Nevertheless, diagnostic tests are still the best way to confirm CVL in sick dogs and should not be totally replaced by diagnosis based only on clinical signs.

Appropriate CVL diagnosis is a big challenge in endemic areas with limited diagnostic resources. We report here that a score based on the clinical signs of sick dogs in a highly endemic region can help CVL diagnosis. Overall, the clinical score proposed had a good performance in discriminating CVL-positive dogs from other sick dogs. Furthermore, some clinical signs were more correlated with CVL positivity, particularly the ocular and cutaneous signs. The results of this study may help veterinarians correctly diagnose CVL in sick dogs; however, further studies with larger samples are needed to confirm and validate this clinical score because this study only examined dogs brought to the veterinary hospital or collected by the Zoonosis Control Center. The higher prevalence of CVL among these dogs could have influenced the positive predictive values of the diagnostic tests.
